# Supplementary figures and images for: Gut microbes predominantly act as living beneficial partners rather than raw nutrients
Source: Sci Rep. 2023 Jul 24;13:11981. doi: 10.1038/s41598-023-38669-7 (PMC10366161; doi:10.1038/s41598-023-38669-7)

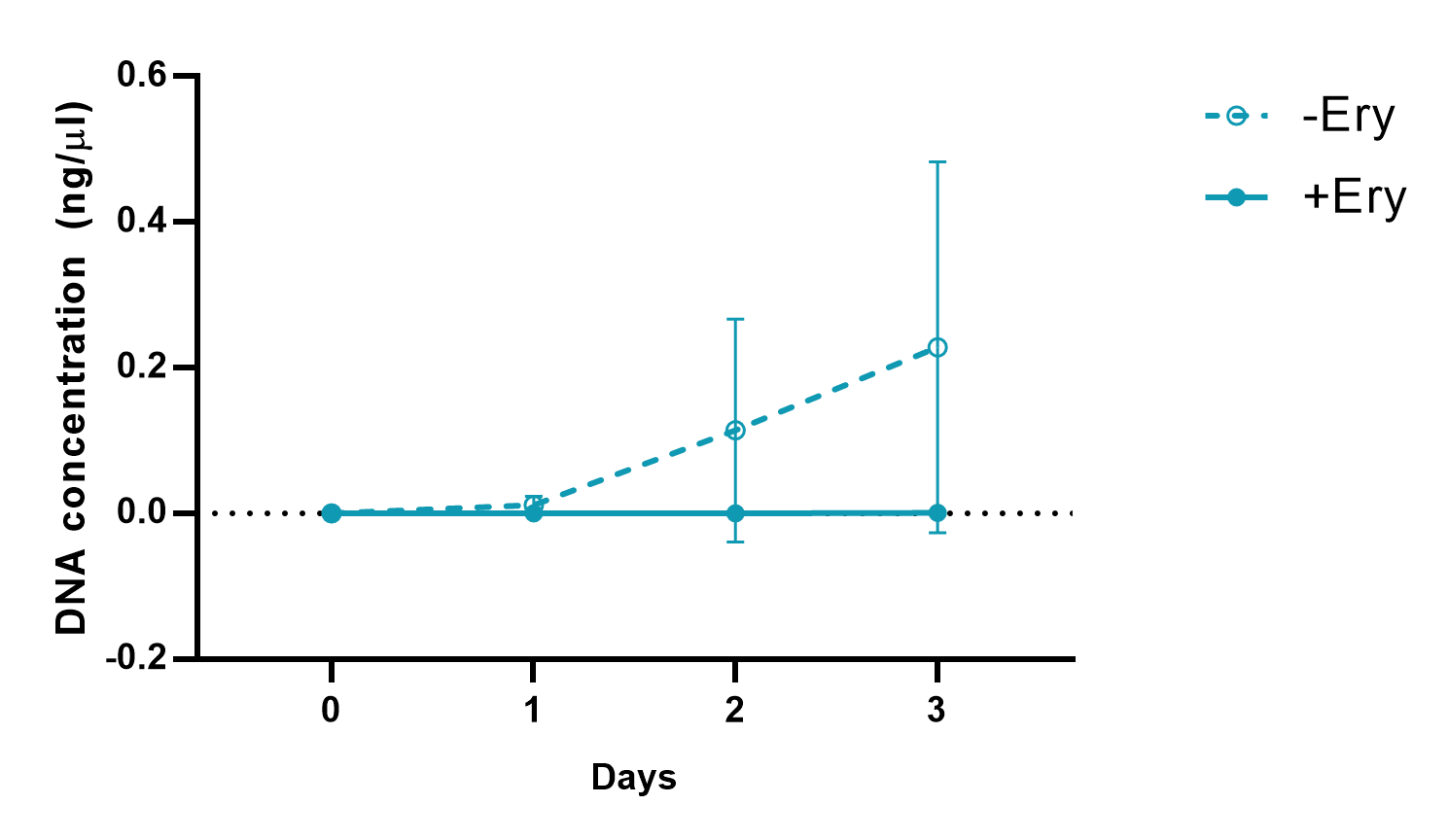

Supplement: Supplementary file 1 — Supplementary Figure S1. [file 41598_2023_38669_MOESM1_ESM.tif]
