# Supplementary material for: Gut microbes predominantly act as living beneficial partners rather than raw nutrients
Source: Sci Rep. 2023 Jul 24;13:11981. doi: 10.1038/s41598-023-38669-7 (PMC10366161; doi:10.1038/s41598-023-38669-7)

Supplementary Figure 2. (A) Larval longitudinal length measured 4 days after inoculation with 1) live bacterial strains on LPD without erythromycin (Live and growing –Ery); 2) live bacterial strains on LPD supplemented with 20µg/ml of erythromycin (Live and stable +Ery); 2) heat -killed bacterial strains on LPD without erythromycin (Dead – HK); 3) UV -treated bacterial strains on LPD without erythromycin (Dead – UV); and 4) PBS on LPD without erythromycin (GF condition). Asterisks illustrate statistically significant difference on pairwise intra-strain comparisons between standard mono-association (Live and growing –Ery) and the respective treatments (including GF) (***: p<0,0001). Center values in the graph represent means and error bars represent SD. (B) Larval longitudinal length measured 7 days after inoculation with PBS on LPD with erythromycin (GF + Ery) and without erythromycin (GF -Ery) (ns: not significant). Center values in the graph represent means and error bars represent SD.

A

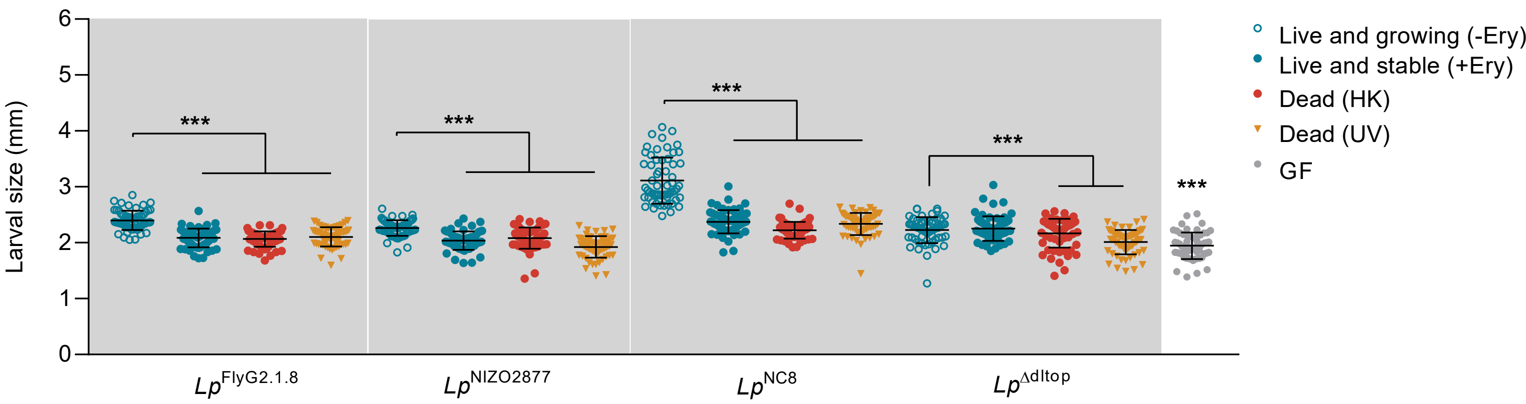

B

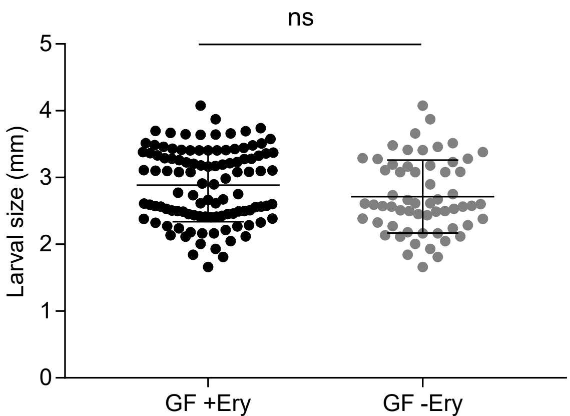

Supplement: Supplementary file 2 — Supplementary Figure S2. [file 41598_2023_38669_MOESM2_ESM.pdf]
