# Supplementary material for: Gut microbes predominantly act as living beneficial partners rather than raw nutrients
Source: Sci Rep. 2023 Jul 24;13:11981. doi: 10.1038/s41598-023-38669-7 (PMC10366161; doi:10.1038/s41598-023-38669-7)

Supplementary Figure 3. (A) Growth trend of the four *L. plantarum* strains monitored over the course of a standard mono-association on LPD. (B) CFUs of the 4 *L. plantarum* strains monitored at day 7 after mono - association on LPD in standard conditions (-Ery), on LPD supplemented with 20 µg/ml of erythromycin with daily addition of live bacterial cells (+ Ery [daily addition]) and on LPD supplemented with 20 µg/ml of erythromycin without daily addition of live bacterial cells (+ Ery [no addition]). Asterisks illustrate statistically significant difference on pairwise intra-strain comparisons between standard mono-association (–Ery) and the respective treatments (***: p<0,0001, ns: not significant). Center values in the graph represent means.

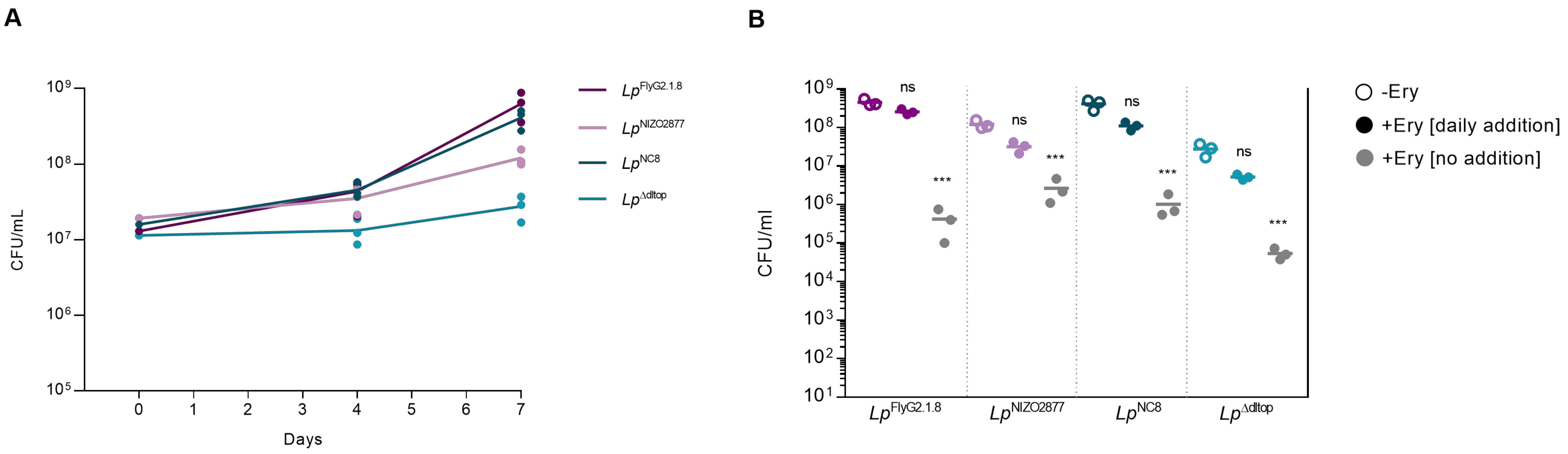

Supplement: Supplementary file 3 — Supplementary Figure S3. [file 41598_2023_38669_MOESM3_ESM.pdf]
