# Supplementary material for: Gut microbes predominantly act as living beneficial partners rather than raw nutrients
Source: Sci Rep. 2023 Jul 24;13:11981. doi: 10.1038/s41598-023-38669-7 (PMC10366161; doi:10.1038/s41598-023-38669-7)

**Supplementary Figure 4**. Larval longitudinal length measured 4 days after inoculation with 1) live bacterial strains on LPD without erythromycin (Live and growing –Ery); 2) daily addition of live bacterial strains on LPD supplemented with 20µg/ml of erythromycin (Live and stable +Ery); 3) daily addition of heat -killed bacterial strains on LPD without erythromycin (Dead -Ery); 4) daily addition of heat-killed bacterial strains on LPD supplemented with 20µg/ml of erythromycin (Dead +Ery) and 5) PBS on LPD without erythromycin (GF condition). Different letters indicate statistically significant differences at $p < 0.05$. Center values in the graph represent means and error bars represent SD.

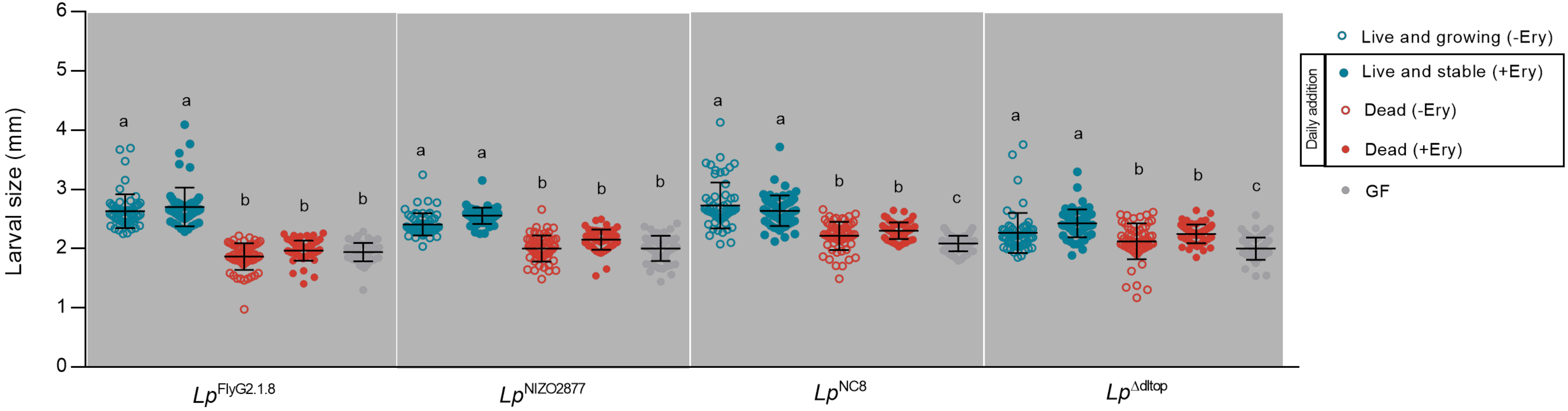

Supplement: Supplementary file 4 — Supplementary Figure S4. [file 41598_2023_38669_MOESM4_ESM.pdf]
