# Supplementary material for: Gut microbes predominantly act as living beneficial partners rather than raw nutrients
Source: Sci Rep. 2023 Jul 24;13:11981. doi: 10.1038/s41598-023-38669-7 (PMC10366161; doi:10.1038/s41598-023-38669-7)

Supplementary Figure 5. Variable importance obtained through random forest analysis for each bacterial feature in promoting larval growth at day 7 after *L. plantarum* $^{FlyG2.1.8}$ (A), *L. plantarum* ^{NIZO2877} (B), *L. plantarum*^{NC8} (C) and *L. plantarum*$^{\Delta dltop}$ (D) association.

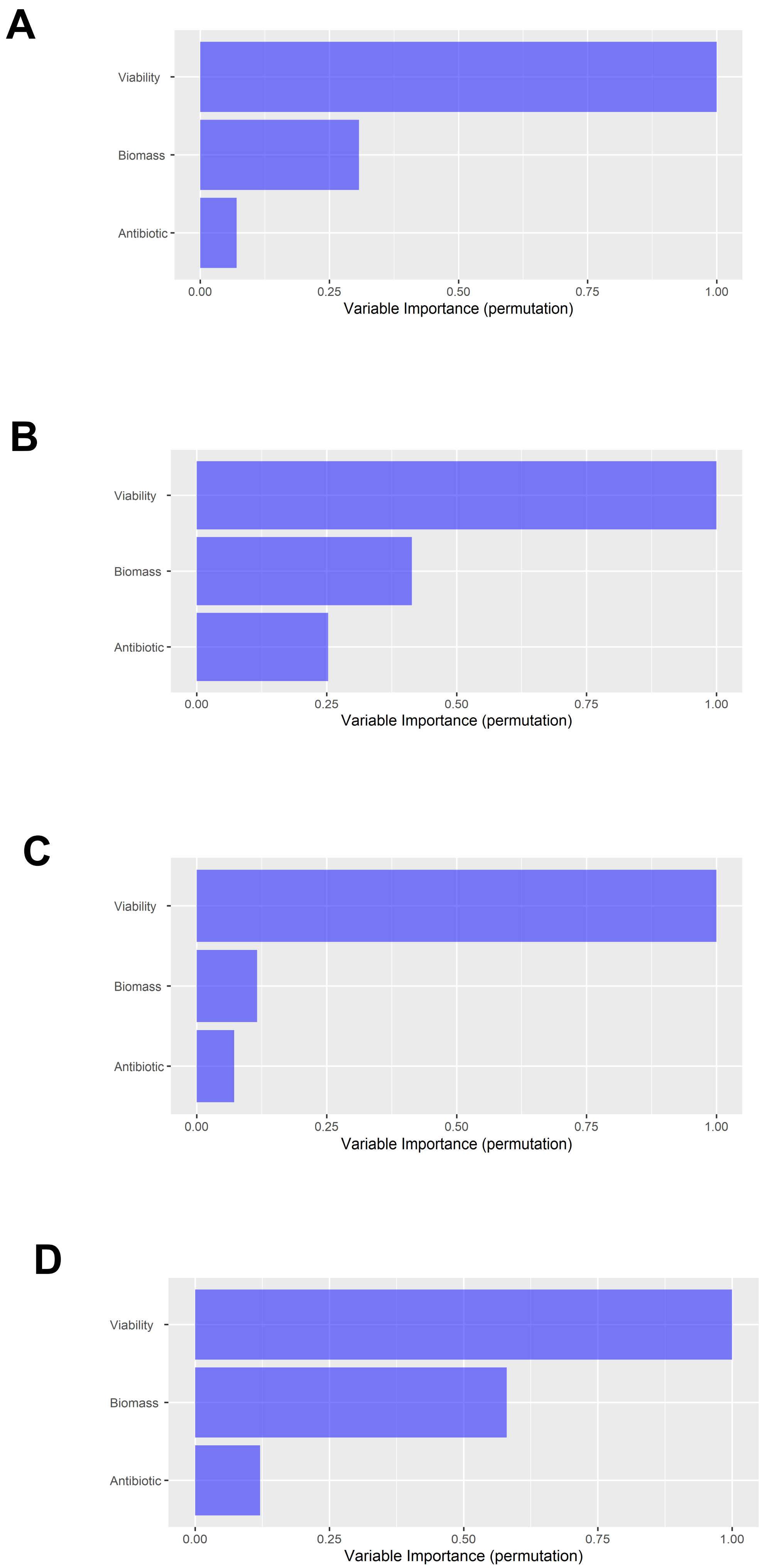

Supplement: Supplementary file 5 — Supplementary Figure S5. [file 41598_2023_38669_MOESM5_ESM.pdf]
